# The role of phospholipid molecular species in determining the physical properties of yeast membranes

**DOI:** 10.1002/1873-3468.12944

**Published:** 2017-12-29

**Authors:** Mike F. Renne, Anton I. P. M. de Kroon

**Affiliations:** ^1^ Membrane Biochemistry & Biophysics Department of Chemistry Bijvoet Center for Biomolecular Research & Institute of Biomembranes Utrecht University the Netherlands

**Keywords:** membrane fluidity, phospholipid molecular species, phospholipid properties

## Abstract

In most eukaryotes, including *Saccharomyces cerevisiae*, glycerophospholipids are the main membrane lipid constituents. Besides serving as general membrane ‘building blocks’, glycerophospholipids play an important role in determining the physical properties of the membrane, which are crucial for proper membrane function. To ensure optimal physical properties, membrane glycerophospholipid composition and synthesis are tightly regulated. This review will summarize our current knowledge of factors and processes determining the membrane glycerophospholipid composition of the reference eukaryote *S. cerevisiae* at the level of molecular species. Extrapolating from relevant model membrane data, we also discuss how modulation of the molecular species composition can regulate membrane physical properties.

## Abbreviations

**DGAT**, diacylglycerol acyltransferase

**FAS**, fatty acid synthase

**GPAT**, glycerol‐3‐phosphate acyltransferase

**GPC**, glycerophosphocholine

**LPAAT**, lysoPA acyltransferase

**PL**, phospholipid

**SFA**, saturated fatty acid

**UFA**, unsaturated fatty acid

Biological membranes are composed of a complex mixture of lipid molecules, in which proteins are embedded. Technological advances over the last decade, especially in the field of mass spectrometry, have allowed detailed analysis of the cellular lipidome, revealing thousands of different lipid molecular species 1. Furthermore, the membranes within the cell were found to differ largely in lipid composition, and the lipid composition is tightly regulated 2, 3. The latter strongly indicates that membrane lipids serve more functions than just as ‘building blocks’ of the membrane matrix. The findings that aberrant lipid composition causes endoplasmic reticulum (ER) stress and triggers stress response signaling further support the important role of lipids in membrane functioning 4. In addition, aberrant lipid metabolism has been associated with the pathology of several diseases, including the hepatic stress observed in obesity 5, fatty acid‐induced lipotoxicity 6, and the promotion of nonalcoholic fatty liver disease 7.

The proper (local) membrane lipid composition is important for organization and dynamics of membranes to allow processes such as budding, membrane trafficking, and fusion and fission events 1, the formation of domains 8, and (transient) formation of protein–protein and protein–lipid complexes. In addition, lipid composition is important for the proper embedding of membrane proteins 9, 10. The lipids surrounding a membrane protein are crucial for its structure and function, either because of direct interaction between a specific lipid and the protein 9 or because of the physical properties of the membrane matrix surrounding the protein, e.g., fluidity, thickness, intrinsic curvature, or lateral pressure profile.

The membrane lipids of the budding yeast *Saccharomyces cerevisiae* can be divided into three different families, based on their molecular structure: glycerophospholipids (or phospholipids in short; PL), sterols, and sphingolipids. Phospholipids make up most of the lipids of the membrane bilayer and therefore play an important role in determining the physico‐chemical properties of the membrane. This review will focus on the role of bulk phospholipids in determining membrane physico‐chemical parameters, with emphasis on the effects of acyl chain and PL class composition, and using the eukaryote baker's yeast as a reference. Research on membrane lipid housekeeping in yeast has been pivotal in the understanding of lipid homeostasis. We will first briefly introduce phospholipid biosynthesis in yeast (section Membrane phospholipid biosynthesis). Subsequently factors determining (the regulation of) membrane acyl chain composition will be discussed, including the role of substrate specificities of phospholipid biosynthetic enzymes (section Processes regulating acyl chain composition and PL molecular species profile). Next, we will review biophysical data from relevant model system studies, and discuss their implications for membrane physico‐chemical parameters (section Phospholipid molecular species profile determines physical properties of the membrane; insights from biophysical studies). Finally, we will discuss some open questions in our understanding of the regulation of membrane lipid composition in maintaining the membrane physical properties (section Conclusions and perspectives).

## Membrane phospholipid biosynthesis

The phospholipid composition of yeast cells is determined by lipid metabolic processes. Lipid composition and metabolism is highly dependent on culture conditions, including temperature (section Processes regulating acyl chain composition and PL molecular species profile), growth phase 11, 12, carbon source 12, 13, the use of rich‐ or minimal media 12 and the presence of lipid precursors such as inositol 14, therefore care must be taken when comparing data from different studies.

Here, we provide a brief overview of phospholipid biosynthesis. For more general reviews regarding the biosynthesis of phospholipids in yeast and its regulation, the reader is referred to other recent reviews 15, 16, 17.

### Acyl chain biosynthesis, desaturation and elongation

The bulk synthesis of acyl chains (or fatty acids) starts with acetyl‐CoA (C_2_) that is converted into malonyl‐CoA (C_3_) by the acetyl‐CoA carboxylase Acc1p, which is the rate limiting step in acyl‐CoA biosynthesis 18. Malonyl‐CoA is then used as a C_2_ donor by the fatty acid synthase (FAS) complex consisting of Fas1p and Fas2p, in elongating the growing acyl chain starting from acetyl‐CoA, yielding the main yeast acyl‐CoAs C16:0 and C18:0 and minor amounts of C10:0, C12:0, and C14:0 18. The acyl‐CoA elongases Elo1p, Elo2p, and Elo3p together with Ifa38p, Phs1p, and Tsc13p in the ER can elongate C14:0‐CoA and C16:0‐CoA to C18:0‐CoA and further up to C26:0 18.

C16:0‐CoA, C18:0‐CoA, and C14:0‐CoA to a minor extent can be desaturated by the Δ^9^‐desaturase Ole1p to C16:1Δ^9^‐CoA, C18:1Δ^9^‐CoA, and C14:1Δ^9^‐CoA, respectively, all of which are in *cis*‐conformation 19. It has been shown that C14:1Δ^9^‐CoA can be elongated to C16:1Δ^11^‐CoA 20. The subsequent Elo1p‐dependent elongation of C16:1Δ^11^ to C18:1Δ^13^ was observed when cells were grown in C14:1Δ^9^‐supplemented media, but even under these conditions C18:1Δ^13^ was only a minor species (0.5%) 20. Furthermore, C16:1Δ^9^‐CoA can be elongated to C18:1Δ^11^‐CoA, also in an Elo1p‐dependent manner 20, 21.

Triglycerides have been proposed to play a role in fatty acid detoxification by storing toxic acyl chains 22, 23. As the level of C18:1Δ^11^ was increased from ± 2.5% to over 10% in a mutant deficient in neutral lipid synthesis compared to the wild‐type, the elongation of C16:1Δ^9^‐CoA to C18:1Δ^11^‐CoA was suggested to be relevant for protecting against lipotoxic effects 21. This was supported by the fact that the neutral lipid deficient mutant was inviable on media containing exogenous C16:1Δ^9^ or C18:1Δ^9^, but not on media containing exogenous C18:1Δ^11^ 21.

### Glycerophospholipid biosynthesis

**Figure 1:**
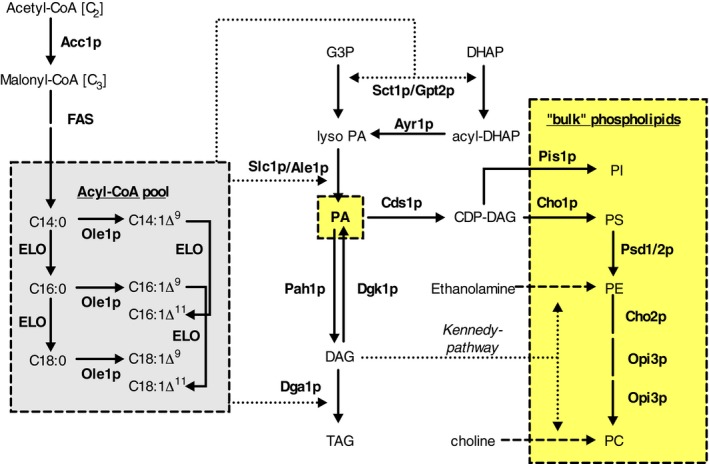
Overview of the main phospholipid biosynthetic pathways in yeast. Enzymes involved in the synthesis of bulk PLs are indicated (in bold). ELO indicates elongation processes by Elo1p and/or Elo2p. The PS‐decarboxylation step (Psd1/2p) is the only key step not localized to ER or cytosol.

The precursor lipid class for all phospholipid classes in yeast is phosphatidic acid (PA), which is synthesized from the chiral water‐soluble precursor *sn‐*glycerol‐3‐phosphate (D‐glycerol‐1‐phosphate; G3P) in the ER (Fig. 1). The G3P acyltransferases (GPATs) Sct1p and Gpt2p acylate G3P to *sn*‐1‐acyl‐G3P or lysoPA using acyl‐CoA as acyl chain donor 24. Sct1p and Gpt2p can also use dihydroxyacetone phosphate (3‐hydroxy‐2‐oxopropyl phosphate; DHAP) as a substrate to produce the pro‐chiral acyl‐DHAP which is reduced to the chiral lysoPA by Ayr1p 25. Subsequently, the lysoPA acyltransferases (LPAATs) Slc1p, Ale1p, Ict1p, and Loa1p can acylate lysoPA at the *sn‐*2 position to form PA 26, 27, 28. As the deletion of *SLC1* and *ALE1* is synthetically lethal, Slc1p and Ale1p are considered the major LPAATs 26.

PA can be either dephosphorylated to diacylglycerol (DAG) by the PA hydrolase Pah1p (in the ER) 29 or activated to CDP‐DAG by Cds1p in the ER or by Tam41p in mitochondria 30, 31. CDP‐DAG is converted into phosphatidylserine (PS) by Cho1p 32, or phosphatidylinositol (PI) by Pis1p 33, both localized in the ER. In the mitochondria, CDP‐DAG is used as precursor for the synthesis of *sn*‐1‐phosphatidylglycerol‐3‐phosphate by Pgs1p 34, which is dephosphorylated to phosphatidylglycerol by Gep4p 35 and subsequently combined with a second CDP‐DAG molecule by Crd1p to form the mitochondrial lipid cardiolipin (CL) 36, 37, 38. PS can be decarboxylated to phosphatidylethanolamine (PE) by the mitochondrial Psd1p or the endosomal Psd2p, both of which require transport of PS from the ER 39. The produced PE can return to the ER to be methylated by Cho2p to monomethyl‐PE, which is subsequently methylated to dimethyl‐PE and PC by Opi3p 40.

DAG is the lipid precursor for an alternative route of PE and PC synthesis *via* the CDP‐ethanolamine and CDP‐choline pathway, respectively, also known as the Kennedy pathway 41. In this pathway, the water‐soluble lipid head group precursors ethanolamine and choline are phosphorylated to phosphoethanolamine and phosphocholine by Eki1p and Cki1p, respectively, and subsequently activated to CDP‐ethanolamine and CDP‐choline by Ect1p and Pct1p. The CDP‐activated head group moieties can react with DAG to form PE and PC in reactions catalyzed by Ept1p and Cpt1p, respectively, which are localized in the ER and/or Golgi apparatus 42.

DAG is also the precursor for the synthesis of the storage lipid triacylglycerol (TAG) by the diacylglycerol acyltransferase (DGAT) Dga1p, which uses acyl‐CoA as acyl chain donor, or by Lro1p that uses a phospholipid as acyl chain donor 43. DAG can also be phosphorylated by the DAG kinase Dgk1p to yield PA 44.

## Processes regulating acyl chain composition and PL molecular species profile

**Figure 2:**
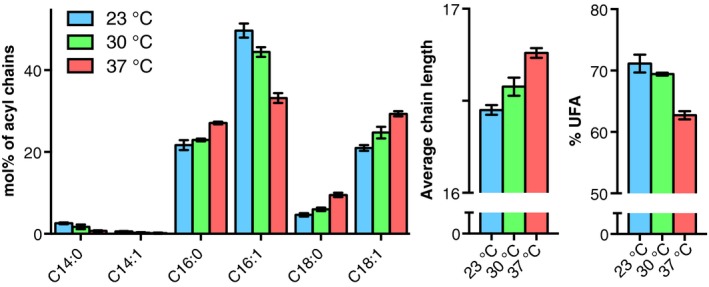
Homeoviscous adaptation in yeast: acyl chain composition at different growth temperatures. Wild‐type yeast cells (strain BY4741) were grown in synthetic defined glucose medium‐ to mid‐log phase at the indicated temperature. Total lipid extracts were prepared, transesterified to fatty acid methyl esters, and analyzed by GC‐FID (M. F. Renne and A. I. P.M. de Kroon, unpublished data). Data are presented as mean values ± SD (*n* = 3).

As a poikilothermic organism, yeast adapts its membrane lipid composition in response to altered growth temperatures in a process known as homeoviscous adaptation 45, 46. Upon a switch to higher growth temperature, the average acyl chain length is increased and the level of unsaturated acyl chains (UFA), mainly C16:1, decreases (Fig. 2; see also Refs 46, 47). In terms of PL molecular species, the content of monounsaturated phospholipids is gradually increased at the expense of diunsaturated phospholipids, which is accompanied by rises in the levels of the major bilayer preferring lipid classes PC and PI at the expense of PE 12. These changes are thought to be driven by the regulation of the expression of lipid biosynthetic genes and of the activity of enzymes, and the consequent alterations of fluxes through the lipid biosynthetic pathways.

### Fatty acid synthesis affects acyl chain length

The acyl chain composition of newly synthesized membrane phospholipids is mainly determined by the extent of desaturation and elongation of the acyl‐CoA pool. The length of the acyl‐CoA species in the acyl‐CoA pool may be regulated by the FAS complex, which was shown to produce different ratios of C16:0‐ to C18:0‐CoA *in vitro* upon temperature variation 48. In addition, the activity of the acetyl‐CoA carboxylase encoded by the *ACC1* gene affects acyl chain length, as was indicated by *in vitro* experiments on fatty acid synthesis in which the concentration of its product malonyl‐CoA was varied 49, and further supported by a thermosensitive and a cold sensitive *ACC1* mutant that shows a higher C_16_/C_18_ ratio at the nonpermissive temperature 50, 51. Recently, the regulation of the activity of Acc1p by phosphorylation has been shown to regulate the ratio of C_16_‐to‐C_18_ acyl chains in the membrane lipids 52.

### Regulation of Ole1p activity

It is widely accepted that the regulation of acyl‐CoA desaturation by Ole1p plays a major role in homeoviscous adaptation in yeast. The activity of Ole1p is regulated at the level of transcription of the *OLE1* gene, which is responsive to temperature, carbon source, and supplementation of exogenous fatty acids 46. The expression of *OLE1* is regulated by the transcription factors Spt23p and Mga2p, which are both synthesized as 120 kDa proteins (p120) and form homodimers residing in the ER membrane 53, 54. Upon activation, Spt23p and Mga2p are ubiquitinylated and subsequently cleaved in a proteasome‐dependent manner to release 90 kDa (p90) effector proteins. The p90 proteins travel to the nucleus and facilitate *OLE1* expression. The deletion of both *SPT23* and *MGA2* is synthetically lethal. Single *spt23*Δ mutants show no alterations in Ole1p levels, but *mga2*Δ have lower Ole1p levels and therefore lower levels of UFA chains implicating Mga2p as the major player in Ole1p regulation 55.

Recently Mga2p dimers were proposed to sense lipid packing *via* their transmembrane helices 56. The authors propose the aromatic residues on the transmembrane helices to face ‘outwards’ to the lipid acyl chains in a fluid environment, and rotate ‘inwards’ towards the monomer–monomer interface in a more rigid environment, due to the tighter packing of the lipid acyl chains, which disfavors the insertion of the aromatic residues into the acyl chain region. When the membrane is fluid the ubiquitination sites of the Mga2p dimer are sequestered. When the membrane lipids are tightly packed, the rotation mechanism exposes the ubiquitination site on both Mga2p monomers, enabling processing by the proteasome and release of p90 56. A similar mechanism likely applies to Spt23p, as the transmembrane helices of Spt23p and Mga2p have 86% sequence similarity 57.

### Substrate specificity of acyltransferases

The substrate specificity of lipid biosynthetic enzymes determines the molecular species profile of synthesized lipids and may have a role in determining the phospholipid molecular species profile (see section Substrate specificity of PL biosynthetic pathways). Pulse labeling strategies in various yeast mutants, *in vitro* enzyme assays, and changes in the lipidome of deletion and overexpression mutants have been used to deduce substrate specificities at the acyl chain level.

Whereas the GPAT Gpt2p was shown to have little substrate preference *in vitro* (except for slightly reduced activity towards C18:0‐CoA), its paralog Sct1p was shown to exhibit a strong preference for C16:0‐CoA and C16:1‐CoA 24. *In vivo*, the deletion of *SCT1* was shown to decrease the C16:0 levels in the four major phospholipids PC, PE, PI, and PS, with the largest effect on PC. Interestingly, deletion of *GPT2* hardly affects the acyl chain composition of PC, PI, and PS, but does increase the C16:0 and C18:1 levels in PE at the expense of C16:1 24. Looking at the total cellular acyl chain composition, De Smet *et al*. 58 showed that deletion of *SCT1* decreases the cellular C16:0 content by almost 50%, whereas overexpression of *SCT1* increases C16:0 levels at the expense of C16:1 and C18:1. Based on these data, it has been hypothesized that Sct1p‐mediated incorporation of C16:0 into PL serves as a sink for C16:0. In line with this hypothesis it was shown that co‐overexpression of *OLE1* rescues the growth defect conferred by *SCT1* overexpression and the accumulation of C16:0, indicating competition between Sct1p and Ole1p for their shared substrate C16:0‐CoA 58. Interestingly, overexpression of *OLE1* increases the level of C18:1 rather than C16:1 58, indicating a preference for C18:0‐ over C16:0‐CoA.

Loss of the LPAAT Ale1p does not significantly affect the PL molecular species profile. In contrast, loss of Slc1p causes a general increase in C_32_ PL at the expense of C_34_ PL 26. A later study showed that this is mainly due to a decrease in C18:1 acyl chains in the PL and an increase in C16:1 with only minor effects on the saturated acyl chains 59, indicating that Slc1p‐mediated incorporation of C18:1‐CoA into PLs may serve as a sink for C18:1.

In a lipidomics study using four double mutants with deletions of *SCT1* or *GPT2* combined with deletion of *SLC1* or *ALE1*, it was shown that deletion of *SLC1* in combination with the deletion of either *SCT1* or *GPT2* has a large effect on the molecular species composition, whereas deletion of *ALE1* in combination with *SCT1* or *GPT2* shows only minor differences 60. As Ale1p, but not Slc1p, is known to have general lysoPL acyltransferase activity 61, 62, 63, 64, these findings were interpreted as indicative for *de novo* synthesis of PL having a more pronounced role in determining the acyl chain at the *sn‐*2 position 60. However, the proposed acyl‐CoA preference of Slc1p *versus* the absence of a clear‐cut acyl‐CoA substrate specificity for Ale1p may also contribute.

The DGAT Dga1p has been proposed to have a substrate specificity for C16:0 and C18:1, based on the observation that yeast cells lacking *DGA1* show a decrease in C16:0 and C18:1 levels which is compensated by an increase in C16:1 65. Care must be taken when interpreting these data, as Dga1p was studied in a background strain deleted for the other neutral lipid synthesis genes (*lro1Δare1Δare2Δ*). Interestingly it was found that in the quadruple mutant *dga1Δlro1Δare1Δare2Δ* the total level of C18:1 is increased compared to wild‐type, which is mainly due to the presence of 7.3% C18:1Δ^11^ 21, 65. The presence of *ARE1* does not decrease the level of C18:1Δ^11^ and *LRO1* or *ARE2* only decrease it to 3.3–3.4%. However, the presence of *DGA1* reduces the C18:1Δ^11^ level to wild‐type (0.1%), consistent with Dga1p being responsible for bulk TAG synthesis.

Besides the direct role in determining the nature of the acyl chains of the synthesized lipid, the substrate specificities of the acyltransferases described above may affect lipid biosynthesis indirectly, by their impact on the composition of the acyl‐CoA pool. By transferring the acyl chains from specific acyl‐CoA species, the acyl‐CoA substrate is no longer accessible to other enzymes catalyzing, e.g., elongation or desaturation. Variation in the activity of enzymes that use bulk amounts of specific acyl‐CoAs, such as Sct1p consuming C16:0‐CoA above, will affect sinks for acyl chains and alter the available acyl‐CoA species by affecting reactions involving acyl‐CoA's by mass action 16. In this context the acyl‐CoA synthetases Faa1‐4p and Fat1p should be mentioned that contribute to the acyl‐CoA pool by converting free fatty acids mobilized from neutral lipids or derived from lipid turnover into acyl‐CoA's 66, 67. Differences in substrate specificity both *in vitro* and *in vivo* have been reported for acyl‐CoA synthetases (reviewed in Ref. 66). The activity and specificity of the acyl‐CoA synthetases may contribute to the composition of the acyl‐CoA pool and thereby influence acyl‐CoA‐dependent metabolic steps.

### Substrate specificity of PL biosynthetic pathways

**Figure 3:**
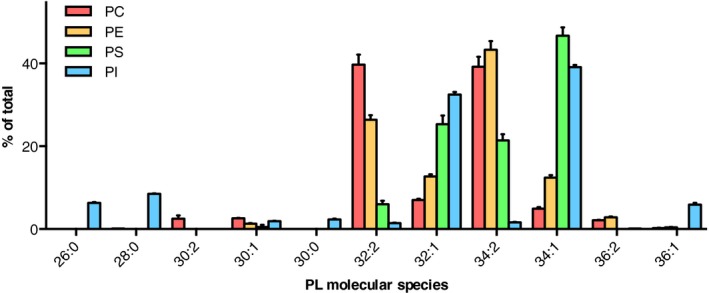
Molecular species profiles of the major membrane phospholipids in yeast. The molecular species of the main glycerophospholipids PC, PE, PS, and PI are shown of wild‐type strain BY4741, cultured to early log phase in synthetic complete glucose medium at 30 °C. Data (mean values with standard deviation; *n* = 3) were taken from the supplementary data of Klose *et al*. 12.

The major yeast lipids PC, PE, PI, and PS have different molecular species profiles (Fig. 3, 12). For PC and PE, 32:2 and 34:2 are the most abundant together amounting to over 75%, followed by 32:1 and 34:1. Interestingly, the disaturated species 32:0 and 34:0 are not detected. The major PS species is 34:1, followed by 32:1 and 34:2. PI is enriched in the disaturated, short acyl chain species 26:0, 28:0, and 30:0 (approx. 17% of total) compared to PC, PE, and PS, in which these are absent. Furthermore, diunsaturated species are virtually absent in PI (less than 5%). The substrate use of the lipid biosynthetic pathways could contribute to the PL‐specific species profiles. The substrate use is determined by the substrate specificity of the individual enzymes and/or the molecular species of the lipid substrate available at their subcellular location. Postsynthetic acyl chain remodeling may further contribute (section Acyl chain remodeling).

Substrate specificity at the level of molecular species of the PA consuming enzymes Cds1p and Pah1p, which produce CDP‐DAG and DAG, respectively, has not (yet) been reported. This also applies to the PI and PS biosynthetic enzymes Pis1p and Cho1p. However, the routes producing the bulk membrane phospholipids PE and PC were reported to show selectivity at the level of molecular species. In the absence of ethanolamine and choline, yeast is mainly dependent on PS decarboxylation for PE synthesis and subsequent methylation to PC. The mitochondrial enzyme Psd1p is the main PS decarboxylase and produces 70–90% of cellular PE 68. Compared to PS, PE and PC are enriched in diunsaturated species (Fig. 3, 12, 69, 70) which could be due to molecular species selectivity of the PS decarboxylase. Alternatively, the species selective conversion of PS to PE could be due to preferential transport of diunsaturated PS species from the ER to mitochondria, as was reported in mammalian cells 71.

The molecular species profiles of PC synthesized *via* either the methylation of PE by Cho2p and Opi3p or by the Kennedy pathway were shown to differ in a stable isotope pulse labeling study using D_13_‐choline and D_3_‐methionine 72. The PE methylation pathway produces mainly 34:2 and 32:2 PC species (approx. 55% and 35% of total, respectively), whereas the CDP‐choline pathway produces a more even distribution of 32:2, 32:1, 34:2, and 34:1 PC species (approx. 40%, 20%, 20%, and 10% of total, respectively). When the Kennedy pathway is inactivated, e.g., by deletion of *PCT1,* the wild‐type PC molecular species profile cannot be produced by biosynthesis alone, and requires acyl chain remodeling of the PE methylation‐derived PC (see section below).

### Acyl chain remodeling

Recently, we reviewed the current knowledge of lipid acyl chain remodeling in yeast 73. Here, we will briefly discuss how the remodeling of PC ensures the proper molecular species profile of this bulk lipid.

PC can be de‐acylated by a phospholipase A to lysoPC, which can subsequently be de‐acylated to glycerophosphocholine (GPC) by a phospholipase B. PC can also be directly de‐acylated to GPC by a phospholipase B. GPC can either be catabolized to choline and glycerol‐3‐phosphate 74, 75, or re‐acylated to yield 1‐acyl‐glycerophosphocholine (lysoPC) 76, e.g., by the recently identified Gpc1p 77. LysoPC can be acylated to PC by a second acyltransferase such as Ale1p 62. The phospholipase D Spo14 can release the head group of PC, yielding PA and choline, which can subsequently be recycled into PC *via* the Kennedy pathway 78.

The de‐acylation of PC and subsequent re‐acylation of lysoPC or GPC was first demonstrated *in vivo* by Wagner & Paltauf using the incorporation of exogenous radiolabeled fatty acids 79. The incorporation of acyl chains was shown to be a very rapid process, observed already after 2 min, however, the incorporation of the label in the lipid pools was not quantified. Using a strain deficient in PE methylation (*cho2∆opi3∆*) with a switchable Kennedy pathway (*P*_*GAL1*_ *‐PCT1*) it was shown that cells can survive on exogenous diC_8_‐PC in the absence of active PC synthesis. Moreover, diC_8_‐PC was shown to be remodeled to the regular PC species such as 32:2, 32:1, 34:2, and 34:1. However, cell growth is delayed and the content of PC is diminished to less than 10% of total compared to over 40% in the wild‐type, indicating that the uptake of diC_8_‐PC followed by PC remodeling is not sufficiently efficient to sustain proper PC levels and optimal cell growth 80.

As mentioned above, in the *pct1Δ* background newly synthesized PC is mainly diunsaturated as in wild‐type and contains virtually no monounsaturated species. However, the steady‐state PC profile does contain monounsaturated species 72 indicating a role for remodeling of PC. This was further supported by the finding that the *sn*‐1 position of newly synthesized PC contains almost exclusively C16:1, whereas steady‐state PC contains C16:0, C16:1, C18:0, or C18:1 at the *sn*‐1 position 72. A follow‐up pulse‐chase study in the *pct1∆* background using the overexpression of *SCT1* to increase the level of saturated acyl chains, revealed that the monounsaturated PC species are increased to nearly steady‐state levels at the expense of diunsaturated PC species in approximately 1 doubling time, involving approximately 40% of the newly synthesized PC molecular species 81. Deletion of the phospholipase B encoding gene *PLB1* was shown to slow down this process, indicating a remodeling mechanism *via* the turnover of PC to GPC. Subsequent acylation of GPC to lysoPC by Gpc1p 77, followed by acylation to PC by Ale1p 76, 82 are likely major contributors to this process.

## Phospholipid molecular species profile determines physical properties of the membrane; insights from biophysical studies

Understanding how physico‐chemical properties of membrane lipids affect membrane properties is key to understanding the importance of the phospholipid molecular species profile for maintaining membrane function *in vivo*. Biophysical studies on model membranes consisting of (mixtures of) well‐defined synthetic lipids or purified lipid extracts have provided much information on how lipid physico‐chemical properties determine membrane physical parameters such as fluidity, bilayer thickness, intrinsic curvature, and lateral pressure profile. It is important to note that most of these parameters are interrelated and therefore a single parameter cannot be modified without affecting the other parameters to some extent. Nevertheless, these studies have enormously contributed to our understanding of the role of lipids in the complex environment of a biological membrane. In the following, we will focus on the various ways in which the phospholipid molecular species profile can affect membrane physical properties and we will discuss possible implications with respect to regulation of membrane lipid composition *in vivo*.

### Membrane fluidity

Membrane fluidity is probably the most extensively studied physical property of membranes, both *in vitro* and *in vivo*. Depending on the temperature, a lipid bilayer can exist in different phases. The two major, biologically relevant phases are the *liquid crystalline phase* (*L*_α_), a fluid and disordered phase, and the *gel phase* (*L*_β_), which forms at lower temperature and is a more ordered and rigid phase. The phase transition temperature (*T*_m_) from the gel to the liquid crystalline phase has been determined for many different lipids, using model membranes composed of pure lipid molecular species.

#### Role of acyl chain composition

**Figure 4:**
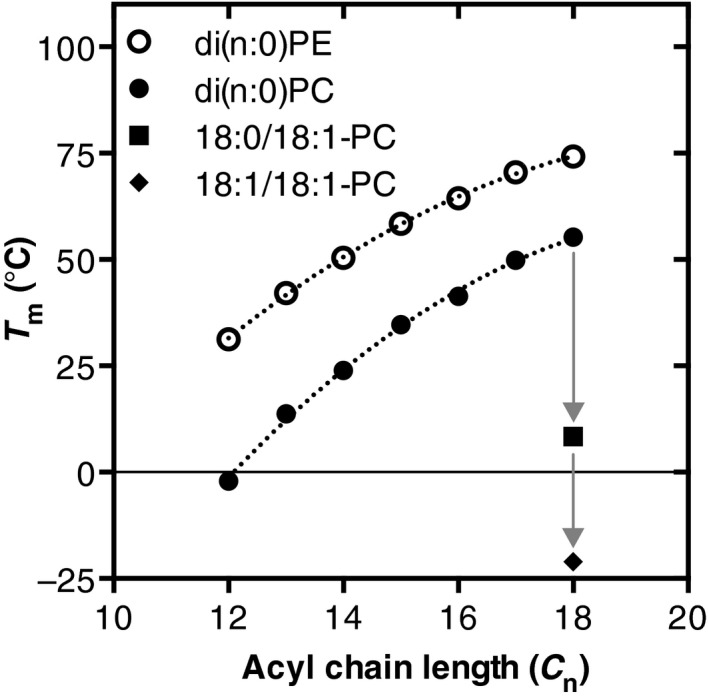
Effect of acyl chain length and unsaturation on the gel‐to‐liquid crystalline phase transition temperature (*T*_m_) of PC and PE. *T*_m_ of completely saturated di(n:0), monounsaturated (n:0)(n:1) and diunsaturated di(n:1) PE and PC species with acyl chains containing the same number of carbons, *n*. Grey arrows highlight the differences in *T*_m_ between saturated, monounsaturated, and diunsaturated PC species. Data were taken from 84 for di(n:0)PC, 112 for (n:0)(n:1)PC, 113 for di(n:1)PC, and 114 for di(n:0)PE. All data were obtained by differential scanning calorimetry of lipid dispersions prepared in water. Curves were added to guide the eye.

**Figure 5:**
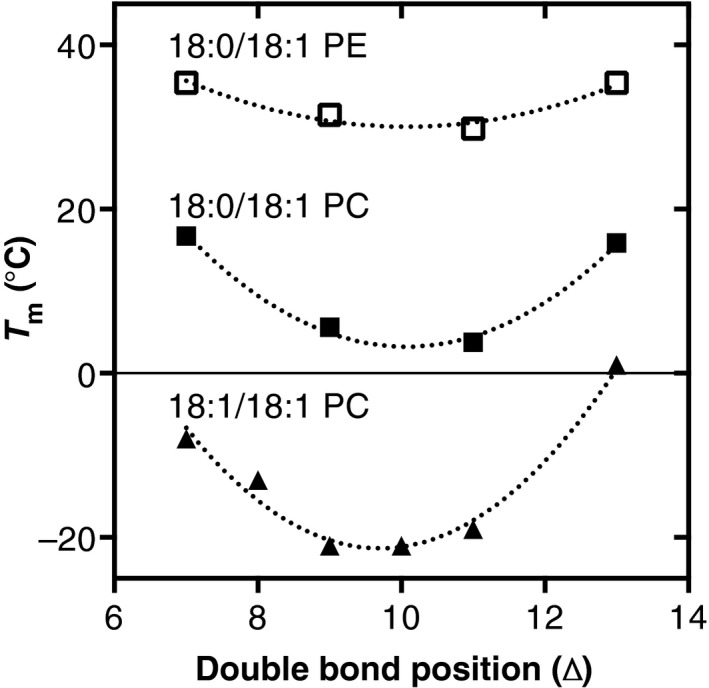
The effect of the position of the *cis‐*double bond (Δ‐location) on the gel‐to‐liquid crystalline phase transition (*T*_m_) of PE and PC species. All data were obtained by differential scanning calorimetry. Representative data were taken from 113 for 18:1/18:1 PC, from 115 for 18:0/18:1 PC, and 116 for 18:0/18:1 PE. Curves were fitted to guide the eye.

The fluidity of a membrane at a given temperature is mainly determined by the acyl chain composition 83. The *T*_m_ increases with increasing lipid acyl chain length 84 and decreases with the introduction of *cis‐*double bonds in the acyl chains 85 (Fig. 4). The decrease in *T*_m_ is larger going from a completely saturated lipid species to a monounsaturated species than from a mono‐ to diunsaturated species (Fig. 4, compare grey arrows. For unsaturated PL species, the location of the *cis‐*double bond also affects *T*_m_ (Fig. 5). Yeast acyl chains comprise mainly Δ^9^ UFAs with only minor amounts of Δ^11^ UFAs (see section Acyl chain biosynthesis, desaturation and elongation; 21, 65). As the difference in *T*_m_ between Δ^9^ and Δ^11^ PL species is very small, this most likely will play but a minor role – if any.

The fluid phase is thought to represent the bulk of the membrane lipids. Maintaining the fluidity of the membrane at altered temperatures is accomplished by adaptation of the acyl chain composition in both degree of unsaturation and acyl chain length (Fig. 2, see also section Processes regulating acyl chain composition and PL molecular species profile). The decreased acyl chain length and increased unsaturation observed at lower growth temperatures are in line with the lower *T*_m_ of shorter and more unsaturated PL species as determined in model membranes (Fig. 4).

#### Role of lipid class composition

Membrane fluidity is also dependent on lipid class composition. PE has a smaller head group than PC and can form hydrogen bonds between the positively charged amino head group and the negatively charged phosphate residue of a neighboring lipid 86. Both these properties cause tighter packing of PE and its acyl chains, which leads to a higher *T*_m_ of PE molecular species compared to the corresponding PC species (Fig. 4). Recently, it was shown that the PE‐to‐PC ratio can be adapted to maintain membrane fluidity in insect cells as well as mammalian cells upon varying the cholesterol content 87. Interestingly, yeast cells grown at higher temperature show a decrease in PE content 12, which is in contrast to the higher *T*_m_ of PE compared to PC (Fig. 4). Therefore, this decrease in PE content is likely to balance membrane intrinsic curvature and lateral pressure profile (see below).

#### Difference in effects between *sn*‐regioisomers of phospholipid species

Lipid species are denoted in various ways, depending on the level of molecular species structural information 88. For example, lipids denoted as 32:1 PC species (defined as a PC lipid with two acyl chains of total 32 C atoms and one double bond) comprise many possible molecular species depending on the acyl chain composition and the *sn*‐regioisomers, for example: 14:0/18:1PC *versus* 18:1/14:0PC, where the first fatty acid is esterfied to the *sn‐*1 position and the second one to the *sn‐*2 position on the glycerol backbone of PC 88. The effect of lipid *sn‐*regioisomers on the total molecular species composition in yeast has not received much attention so far. However, biophysical studies using model membranes have shown striking differences between *sn*‐regioisomers, e.g., in their melting temperature (Table 1). Differences in the insertion depth of the *sn*‐1 and *sn*‐2 acyl chains have been shown, where the *sn*‐2 inserts less deeply into the bilayer, leading to different effects of the *sn‐*regioisomers on lipid packing 89.

**Table 1:** Effect of *sn‐*regioisomers on the gel‐to‐liquid crystalline phase transition temperature (*T*_m_). Data were taken from The Handbook of Lipid Bilayers (second edition, 2013) and references therein 120. Tm of lipids with the same acyl chain at the sn‐1 and sn‐2 positions are depicted in bold

	*sn‐*2				
	12:0	14:0	16:0	18:0	18:1
*sn‐*1					
12:0	**−2.1**		21.7	23.3	
14:0		**23.9**	34.9	39.2	−19.1
16:0	11.3	27.5	**41.4**	48.8	−4
18:0	17.5	31	44.5	**55.3**	5.6
18:1			−9	10	**−17.3**

For monounsaturated lipids, in general, the regioisomer with the saturated or longer acyl chain at the *sn*‐1 position has a lower melting temperature compared to the regioisomer with the saturated or longer acyl chain at the *sn*‐2 position. This might explain the strong enrichment of saturated acyl chains at the *sn*‐1 position in yeast PL 79 that may serve as a mechanism to mitigate the effect of these acyl chains on membrane rigidity. To our knowledge, no studies have investigated the regulation of *sn‐*regioisomers in relationship with the regulation of membrane fluidity in yeast.

#### Influence of sterols

The addition of sterols to a phospholipid bilayer diminishes the differences between the fluid and gel phases. By ordering the acyl chains in a liquid crystalline phase, sterols make membranes more rigid. Conversely, in the gel phase, sterols cause disorder of the saturated acyl chains, thus increasing membrane fluidity. Because of decreased differences between the gel phase and liquid crystalline phase at high sterol content, the phase transition is no longer observed 90. In yeast, ergosterol content was shown to differ only slightly in yeast cultures grown at different temperatures 12.

### Membrane thickness

Membrane thickness is determined by the effective length of the lipid acyl chains, which is mainly determined by the number of carbon atoms in the acyl chain but can be modulated by several factors. First, the bilayer thickness of a lipid in a gel phase will be more ordered than in a fluid phase, and hence the bilayer will be thinner in a fluid phase membrane. Similarly, bilayer thickness of a membrane composed of unsaturated lipids will be slightly less than that of a bilayer consisting of the corresponding saturated lipid. Furthermore, for lipids in the fluid phase, bilayer thickness will decrease with increasing temperature due to increased acyl chain disorder. Consistently, sterols generally increase membrane thickness because of their stretching/ordering effects on especially saturated acyl chains in the fluid phase.

In yeast cells the plasma membrane is the thickest membrane and accordingly it is enriched in sterols and saturated lipid species 2, 70. Consequently, plasma membrane‐localized proteins have significantly longer transmembrane segments than Golgi or ER resident membrane proteins 91. Matching of transmembrane helix length to the bilayer thickness (hydrophobic matching) has been proposed as a mechanism for sorting and retention of proteins 92.

The close relationship between bilayer thickness and membrane fluidity is reflected in the mechanism proposed for the regulation of membrane fluidity by the bacterial thermosensor DesK 93, 94. When bacteria are exposed to lower temperatures, DesK is activated and downstream signaling leads to expression of a desaturase which increases membrane lipid unsaturation 94. DesK has been proposed to sense the temperature‐induced decrease in membrane fluidity as an increase in membrane thickness, based on studies using a “minimal sensor” mutant of DesK 93. Recently, the yeast ER stress sensor Ire1p was proposed to sense the physical state of the membrane by sensing membrane thickness 95. The authors present evidence that Ire1p locally compresses the membrane bilayer. The higher energetic cost of bilayer compression resulting from increased bilayer thickness induced by aberrant lipid composition is proposed to drive Ire1p dimerization 95.

### Lipid polymorphism and membrane intrinsic curvature

**Figure 6:**
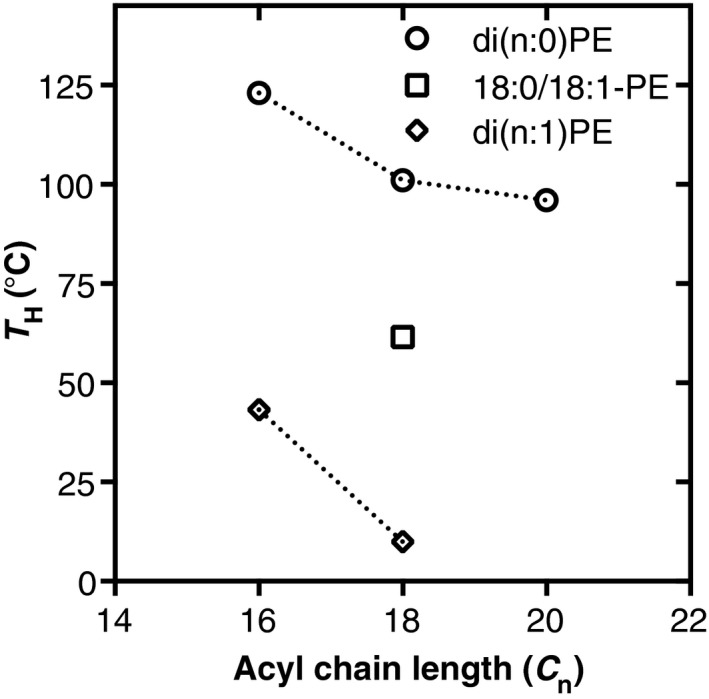
Effect of acyl chain composition on the bilayer‐to‐hexagonal phase transition temperature (*T*_H_) of PE. All data were obtained by differential scanning calorimetry. Representative data were taken from 117 for di(n:0)PE, 118 for 18:0/18:1‐PE, and 119 for di(n:1)PE.

Not all phospholipid species, when hydrated, assemble in bilayers. Indeed, there are many examples of lipids that have a preference for assembling as nonbilayer phases, e.g., an inverted hexagonal phase or micelle. The phase preference of a lipid can be rationalized by the simple, but effective ‘shape structure’ concept 96, in which the ratio of the cross‐sectional area of the lipid head group over the cross‐sectional area of the acyl chain region determines which phase the lipids adopt. When the cross‐sectional area of the lipid head group is close to that of the acyl chain region, the molecule is cylindrical and prefers to form bilayers. In type I lipids, such as various lysoPLs, the head group cross‐sectional area is large compared to that of the acyl chain, giving them the tendency to form positively curved structures. Therefore, type I lipids assemble as micelles in aqueous environment. Conversely, type II lipids, e.g., most diunsaturated PE and PA molecular species, preferably form inverted hexagonal phases upon hydration due to their small head group area compared to that of the acyl chains 97. It should be realized that the nature of the acyl chains determines the extent of the nonbilayer preference or intrinsic curvature by influencing the shape of the molecule 98. This is reflected in the bilayer‐to‐hexagonal (*H*_II_) phase transition temperature (*T*_H_; Fig. 6).

A lipid monolayer of type II nonbilayer preferring lipids will have the tendency to form a negatively curved surface (i.e., intrinsic curvature). When mixed with bilayer lipids, type II lipids can be forced to assemble in a bilayer allowing, e.g., mitochondrial membranes to form bilayers despite their very high content of the nonbilayer lipids PE and CL. However, due to the intrinsic curvature of type II lipids, this will induce curvature stress 97. The presence of nonbilayer lipids and possibly the transient formation of nonbilayer structures have been proposed to be important for fusion and fission processes 99.

Yeast cells were proposed to regulate the intrinsic curvature, by modulating the PE molecular species profile 100. Upon depletion of PC in a yeast *cho2opi3* mutant, PE levels increase and PE takes over as the major membrane PL. In parallel, the proportion of monounsaturated PE species increases at the expense of diunsaturated species, and a shortening of the average acyl chain length is observed. As diunsaturated PE species and PE species with longer acyl chains have stronger nonbilayer propensity, as shown by a lower *T*_H_ (Fig. 6), the decrease in these species was interpreted as a mechanism to regulate membrane intrinsic curvature in response to PC depletion. Indeed, PE extracted from yeast cells under PC depleting conditions was found to have a higher *T*_H_, indicative for PE species that are more bilayer compatible. The accompanying shortening of the average chain length of the PE molecular species also decreases the *T*_m_ of the PE, consistent with the adaptation of the PE molecular species also compensating for the decreased membrane fluidity that would result from the increased PE level and decrease in unsaturated PE species (Fig. 4).

### Lateral pressure profile

The intrinsic curvature of a membrane is closely related to the lateral pressure profile of a membrane, arising from the same structural properties of the membrane lipids 99. The lateral pressure profile reflects the depth‐dependent distribution of lateral stress in a membrane 101. The nature of this phenomenon and its relevance for membrane structure and function can be qualitatively understood as follows. The hydrophobic effect causes lipids to come together and form bilayers, giving rise to an attractive force, or negative lateral pressure, between the lipids at the head group–acyl chain interface. At the same time, interactions between neighboring acyl chains in the hydrophobic core and between hydrophilic head groups with bound water molecules will cause steric repulsion, giving rise to a positive lateral pressure in these regions. Together this leads to an inhomogeneous pressure profile along the bilayer normal (i.e., the lateral pressure profile). Importantly, the lateral pressure is net zero in self‐assembled bilayer structures 102.

Upon increasing the ratio of nonbilayer‐to‐bilayer preferring lipids, the (positive) lateral pressure between the head groups is reduced because of the smaller effective head group volume of the nonbilayer lipids. As a consequence, the lipids will pack more tightly, leading to an increase in the lateral pressure between the acyl chains.

The lateral pressure profile of a membrane affects membrane protein structure and stability 103, 104. As the membrane intrinsic curvature and the lateral pressure profile are related properties, the lateral pressure profile is likely to be regulated in a similar manner as the intrinsic curvature and this may affect protein conformation and/or function 105. For example, a protein in a membrane with a high PE content will have more freedom of movement in the head group area due to the locally lower lateral pressure, but will experience higher lateral pressure in the hydrophobic core 99. In addition, the presence of PE with its relatively small head group may promote insertion of hydrophobic molecules into the lipid/water interface 106. Vice versa, insertion of hydrophobic molecules into a bilayer may modulate the lateral pressure profile. For example, the insertion of alcohols in the head group region of a membrane has been suggested to destabilize membrane proteins by increasing the lateral pressure in the head group region and decreasing the lateral pressure in the hydrophobic core 106.

## Conclusions and perspectives

Thorough genetic studies in combination with lipid analysis and lipidomics studies have provided insight into phospholipid biosynthesis and its regulation. However, mechanisms such as the regulation of acyl chain remodeling, the sensing of membrane physico‐chemical properties, and its downstream signaling to the lipid biosynthetic machinery largely remain to be elucidated. Recently, the molecular mechanisms of membrane sensing by the *OLE1* transcriptional regulator Mga2p and the ER stress sensor Ire1p have been elucidated 56, 95, as described above. Whereas Mga2p responds to differences in lateral pressure, Ire1p is proposed to sense bilayer thickness by a mechanism based on membrane compression that is dependent on the amphipathic helix neighboring the transmembrane helix. Membrane binding amphipathic helices occur in many proteins and have been well studied in terms of the membrane physical parameters they recognize and how membrane binding influences their function 107, 108. Furthermore, membrane binding by amphipathic helices can be modulated, e.g., the amphipathic helix of Pah1p is not recruited to PA‐containing membranes upon phosphorylation 109.

Although transcriptional regulation *via* the Henry regulatory circuit controlled by Opi1p sensing the PA level is well characterized for a set of lipid biosynthetic genes containing UAS_INO_ in their promoter 15, regulation at the (post‐)translational level of most lipid biosynthetic enzymes is not understood, with the notable exception of Pah1p 29. Many lipid biosynthetic enzymes are phosphorylated 110. However, the function of phosphorylation in the regulation of, e.g., activity, subcellular localization or degradation is often not known. Furthermore, the kinases and phosphatases involved in the phosphorylation remain to be identified for most enzymes.

Recent advances in lipidome analysis have provided opportunities to identify lipid characteristics that were previously difficult to distinguish, such as double bond localization and *sn*‐regioisomers 111. Integration of these state‐of‐the‐art lipid analysis techniques with stable isotope labeling will enable dynamic lipidomics studies that will help reveal mechanisms in the regulation of PL molecular species profiles. By combining observations from such experiments with current and new data from biophysical studies on synthetic lipids and biological lipid extracts, this approach will provide novel insights into the regulation of key physico‐chemical properties of the membrane such as membrane fluidity.
